# Marine Fish-Derived Lysophosphatidylcholine: Properties, Extraction, Quantification, and Brain Health Application

**DOI:** 10.3390/molecules28073088

**Published:** 2023-03-30

**Authors:** Mirja Kaizer Ahmmed, Mayssa Hachem, Fatema Ahmmed, Ali Rashidinejad, Fatih Oz, Adnan A. Bekhit, Alan Carne, Alaa El-Din A. Bekhit

**Affiliations:** 1 Riddet Institute, Massey University, Private Bag 11 222, Palmerston North 4442, New Zealand; 2 Department of Fishing and Post-Harvest Technology, Faculty of Fisheries, Chattogram Veterinary and Animal Sciences University, Chattogram 4225, Bangladesh; 3 Department of Chemistry and Healthcare Engineering Innovation Center, Khalifa University, Abu Dhabi P.O. Box 127788, United Arab Emirates; 4 Department of Chemistry, University of Otago, Dunedin 9054, New Zealand; 5 Department of Food Engineering, Ataturk University, Yakutiye 25030, Turkey; 6 Allied Health Department, College of Health and Sport Sciences, University of Bahrain, Sakhir 32038, Bahrain; 7 Department of Pharmaceutical Chemistry, Faculty of Pharmacy, University of Alexandria, Alexandria 21521, Egypt; 8 Department of Biochemistry, University of Otago, Dunedin 9054, New Zealand; 9 Department of Food Science, University of Otago, Dunedin 9054, New Zealand

**Keywords:** marine lysophosphatidylcholine (LPC), Mfsd2a transporter, structural properties, cognitive function, neural health, omega-3 supplementation, mental wellness, processing impact, disease management, drug delivery, bioactive compounds

## Abstract

Long-chain omega-3 fatty acids esterified in lysophosphatidylcholine (LPC-omega-3) are the most bioavailable omega-3 fatty acid form and are considered important for brain health. Lysophosphatidylcholine is a hydrolyzed phospholipid that is generated from the action of either phospholipase PLA_1_ or PLA_2_. There are two types of LPC; 1-LPC (where the omega-3 fatty acid at the *sn*-2 position is acylated) and 2-LPC (where the omega-3 fatty acid at the *sn*-1 position is acylated). The 2-LPC type is more highly bioavailable to the brain than the 1-LPC type. Given the biological and health aspects of LPC types, it is important to understand the structure, properties, extraction, quantification, functional role, and effect of the processing of LPC. This review examines various aspects involved in the extraction, characterization, and quantification of LPC. Further, the effects of processing methods on LPC and the potential biological roles of LPC in health and wellbeing are discussed. DHA-rich-LysoPLs, including LPC, can be enzymatically produced using lipases and phospholipases from wide microbial strains, and the highest yields were obtained by Lipozyme RM-IM^®^, Lipozyme TL-IM^®^, and Novozym 435^®^. Terrestrial-based phospholipids generally contain lower levels of long-chain omega-3 PUFAs, and therefore, they are considered less effective in providing the same health benefits as marine-based LPC. Processing (e.g., thermal, fermentation, and freezing) reduces the PL in fish. LPC containing omega-3 PUFA, mainly DHA (C22:6 omega-3) and eicosapentaenoic acid EPA (C20:5 omega-3) play important role in brain development and neuronal cell growth. Additionally, they have been implicated in supporting treatment programs for depression and Alzheimer’s. These activities appear to be facilitated by the acute function of a major facilitator superfamily domain-containing protein 2 (Mfsd2a), expressed in BBB endothelium, as a chief transporter for LPC-DHA uptake to the brain. LPC-based delivery systems also provide the opportunity to improve the properties of some bioactive compounds during storage and absorption. Overall, LPCs have great potential for improving brain health, but their safety and potentially negative effects should also be taken into consideration.

## 1. Introduction

**Figure 1:**
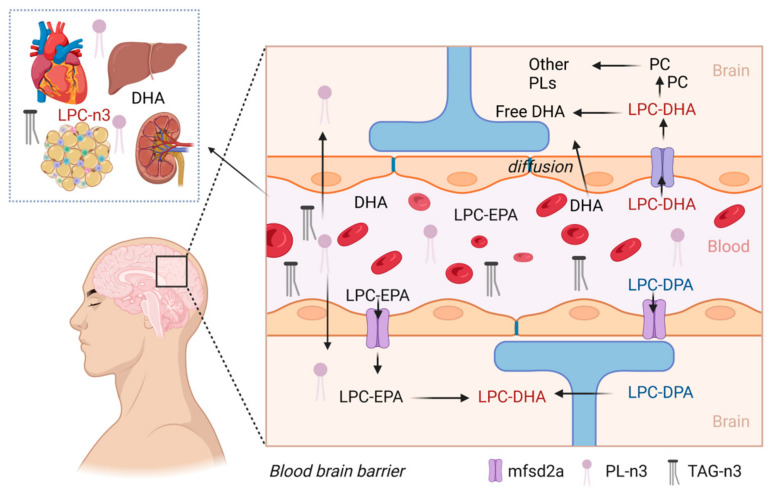
Absorption pathways of different forms of omega-3 fatty acids by the heart, liver, kidney, and brain. Abbreviations: PC = phosphatidylcholine; LPC = lysophosphatidylcholine; EPA = eicosapentaenoic acid; DPA = docosapentaenoic acid; DHA = docosahexaenoic acid; PL = phospholipids; *mfsd2a* = lysolipid transporter.

Phospholipids constitute approximately 60% of the total lipid content of mammalian cells, whereas, in fish, they account for 4–42% of the total lipid, depending on the extraction method, organ, species, and season [1,2]. Phospholipids are vital molecules for cellular homeostasis and are involved in various regulatory and structural functions, as well as being fundamental components of cell membranes. There are more than 1000 different phospholipids found in nature that vary in their chemical structures. Phospholipids consist of phosphorylated alcohol and at least one fatty acid (FA) connected to a glycerol or sphingosine backbone [3,4]. Phospholipids are categorized into different groups, such as phosphatidylcholine, phosphatidylethanolamine, phosphatidylglycerol, phosphatidylinositol, and phosphatidylserine, based on the alcohol structure and/or the number and type of fatty acids (FAs) linked to the glycerol molecule backbone [5]. Due to the presence of polar (head) and non-polar (tail) parts in their structure, phospholipids are described as amphiphilic molecules and exhibit both hydrophobic and hydrophilic features [6]. Long-chain omega-3 polyunsaturated fatty acids (omega-3 PUFAs) such as eicosapentaenoic acid (EPA), docosapentaenoic acid (DPA), and docosahexaenoic acid (DHA) are available in nature either in triacylglycerol (TAG) or phospholipid form [7]. Research has shown that the phospholipid form of omega-3 fatty acids is more bioavailable for the brain, kidneys, liver, and heart compared to the TAG form [3,8]. The TAG form of omega-3 fatty acids is hydrolysed into free fatty acids (FFA) by pancreatic lipases, absorbed by the intestinal mucosa, and transferred into the bloodstream. The FFAs are then deposited into lipoprotein structures known as chylomicrons, which carry the FAs from the small intestine to the blood circulation via a passive diffusion mechanism. TAG-containing chylomicrons (CM-TAG) are mostly absorbed by adipose tissue and the liver, kidney, and heart muscle (Figure 1). CM-TAGs have a preference to be absorbed by adipose tissue, which reduces their bioavailability to other organs such as the brain, eyes, heart, kidney, and liver [9]. On the other hand, the phospholipid form is absorbed in the small intestine as intact micelles and is converted by phospholipase into either a non-esterified fatty acid (NE-DHA) or lysophospholipid form (LPC-DHA) [10]. Lipase or phospholipase cannot break down lysophospholipids, which can be either absorbed by the heart, kidney, liver, or adipose tissue or transported into the brain as LPC-DHA (Figure 1).

Among the phospholipids, lysophosphatidylcholine (LPC) is considered the most bioavailable phospholipid form for the brain as the majority of LPC containing omega-3 PUFAs (longer than 14-carbon) such as LPC oleate, LPC palmitate, LPC-EPA, and LPC-DHA can cross the blood–brain barrier (BBB) via a specific protein coding gene and lysolipid transporter “*mfsd2a*”, enriching the DHA and phospholipid levels in the brain [8,11,12]. The brain has a preference to uptake LPC that has unsaturated fatty acids that are esterified, and not saturated fatty acids [13]. LPC-DHA has been reported to enhance the brain’s DHA content, whereas other forms of omega-3 fatty acids are not efficiently replenished [14,15]. This finding is particularly important since dietary LPC-DHA was found to be more effective than dietary PC-DHA/PC-EPA in improving the behaviour of mice by modulating APOE4 expression, which is associated with age-related cognitive decline and subsequent neurodegenerative disorders [16].

In a rat model, LPC-DHA was found to be preferentially taken up by the brain at a level of 4.5% of the injected radiolabelled LPC-DHA, compared to free DHA that was taken up at only 0.3–0.4% of radiolabelled non-esterified DHA [13]. Although free DHA enters the brain by passive diffusion (Figure 1), it appears that it is not able to enhance the DHA level in the brain, possibly due to being rapidly oxidised [12,17,18]. Recently, several comprehensive reviews have been published that have discussed the structure, properties, sources, extraction, quantification, and health benefits of marine phospholipids [3,7,8,19,20,21,22]. Only a few reviews have focused on the health benefits of lysophospholipids [23,24,25,26], but information on their extraction, properties, quantification, processing effects, and delivery is still limited. This provides an opportunity for future comprehensive reviews to serve as a useful reference for academia and industry.

## 2. Structure and Biosynthesis of LPC

**Figure 2:**
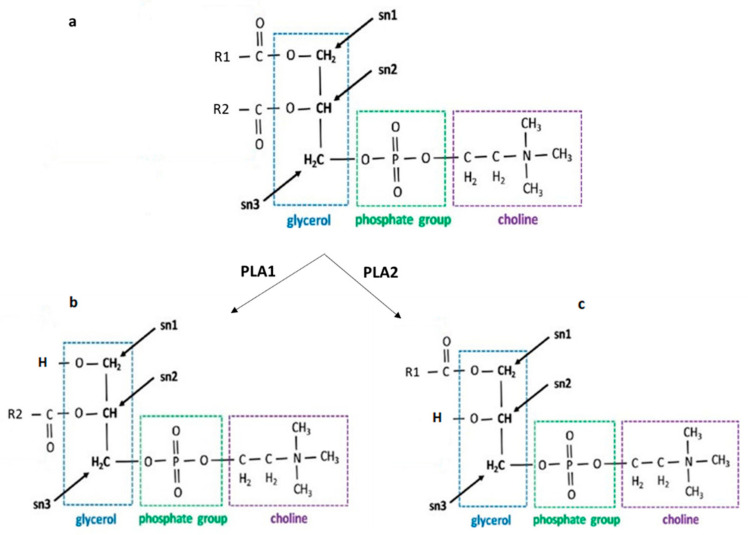
Biosynthesis of LPC through enzymatic hydrolysis of PC. Natural phospholipids (**a**) are hydrolysed by PLA1 and PLA2 to produce 1-LPC (**b**) and 2-LPC (**c**), respectively.

Lysophosphatidylcholine (LPC) is a glycerophospholipid lacking one acyl group, with the free hydroxyl group of the glycerol backbone acylated. In 1-lysophosphotidylcholine (1-LPC), a fatty acid is acylated at position 2 (*sn*-2 position) of the glycerol backbone, whereas in 2-lysophosphotidylcholine (2-LPC), position 1 (*sn*-1 position) is acylated. Figure 2 represents the biosynthesis of 1-LPC and 2-LPC through the enzymatic hydrolysis of PC.

Fatty acids can be saturated (SAFA), monounsaturated (MSAFA), or polyunsaturated fatty acids (PUFA). Mostly, SAFA and MSAFA are acylated at the *sn*-1 position, whereas PUFA are mainly acylated at the *sn*-2 position in LPC [17,18]. 2-LPC is more stable than 1-LPC because of the primary alcohol group at the *sn*-1 position [23,24,25,26]. In vivo, LPC is biosynthesized via the enzymatic hydrolysis of phospholipid (PL), is implicated in PL metabolism, and plays a role as a second messenger, exhibiting numerous biological activities.

For *sn-1* deacylation, 1,3-regioselective lipases (triacylglycerol acylhydrolases, E.C.3.1.1.3) are also very effective catalysts for the hydrolysis of ester bonds that are mainly DHA-rich-PL [27]. 1-LPCs with different FAs (palmitic acid C16:0, arachidonic acid C20:4n-6, linoleic acid C18:2n-6, oleic acid C18:1n-9) acylated at the *sn-2* position were produced from PC through the action of *Rhizoppus arrhizus* [28]. This was achieved through the incubation of 1 to 4 mg of PC with 40,000 IU of the lipase in 500 µL ethyl ether and 500 µL of a borax buffer, pH 6.5. The mixture was agitated at room temperature for 1 h; then, the reaction was terminated by the addition of ethanol. Lipids were extracted with CHCl_3_/CH_3_OH/H_2_O (49/49/2, *v*/*v*/*v*) [28]. The same research group produced 1-LPC with DHA esterified at the *sn-2* position [13], as well as in another subsequent research [29].

Additionally, 1-LPC or 2-LPC enriched in DHA were produced through the action of twelve different lipases on selective DHA-enriched PLs isolated from the microalgae *Isochrysis galbana* [30]. These lipases were obtained from various microorganisms, including *Aspergillus niger*, *Candida rugose*, *Rhizopus Oryzae*, *Penicillium camembertii*, *Mucor javanicus*, *Burkholderia cepacia*, *Penicillium roquefortii*, *Rhizopus niveus*, *Candida antarctica B (immobilised)*, *Thermomyces lanuginose (immobilised)*, and *Rhizomucor miehei (immobilised)*. Microalgal PLs were hydrolysed using lipase at two different concentrations (10 or 20 mg per mL) in 20 mM of a phosphate buffer, pH 7.2 at 40 °C. The reaction was stopped at a specific time between 2 and 26 h, and lipids were extracted in CHCl_3_/ CH_3_OH (2:1 *v*/*v*). Among the microorganisms investigated, *Rhizopus oryzae* and *Mucor javanicus* produced LPC fractions containing DHA with a recovery yield of 85% and 71%, respectively, after a reaction time of 3 h [30].

**Figure 3:**
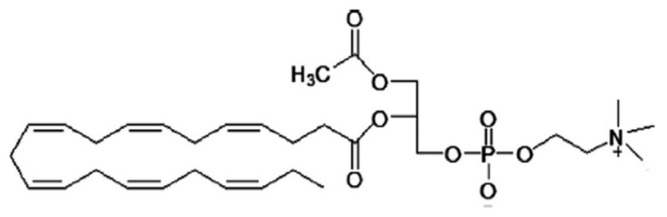
AceDoPC^®^ (1-acetyl, 2-docosahexaenoyl-glycerophosphocholine, AceDoPC^®^) structure [33].

Furthermore, DHA-rich-LysoPLs, including LPC, were enzymatically produced using PLs isolated from the marine microalgae *Isochrysis galbana*, which is considered a natural DHA-rich raw material [24]. The authors suggested that when performing the deacylation of PLs using lipase/phospholipase A_1_, the reaction should be prudently verified in order to fully characterize the reaction products. The key desirable outcome of this reaction is to retain DHA at the *sn-2* position in the produced LPC and to avoid the relocation of the DHA moiety to the *sn-1* position. Several methodologies have been suggested to decrease the incidence of acyl migration during deacylation. These include the usage of a packed bed reactor or adding a borax buffer to the reaction medium. The reaction temperature may likewise be a factor that can influence the reaction and the relocation of DHA. For example, decreasing the reaction temperature from 40 °C to 30 °C during the synthesis of DHA-rich LPLs is reported to reduce DHA migration by 37% [31,32]. More recently, to avoid FA migration from the *sn-2* to the *sn-1* position in LPC-DHA, the *sn-1* position was blocked with an acetyl group, leading to the formation of a phospholipid named 1-acetyl-2-docosahexaenoyl-glycerophosphocholine or AceDoPC^®^ (Figure 3). AceDoPC^®^ is considered to be a stabilised form of LPC-DHA [33,34,35,36].

In addition to the issue of acyl group migration in the LPC issue, the choice of an appropriate lipase for deacylating PC is a challenge. Endothelial lipase (EL) can be used since EL is a PLA_1_ found in the endothelial cells of the blood–brain barrier and is responsible for generating LPC-DHA in plasma. EL has been found to have a hydrolytic specificity towards the polar head of PL, with a preference for PE > PC > PS and a preference for classes comprising DHA in position *sn-2* [37]. Another method for generating Lyso-PL involves the lipase-catalysed acylation of L-α-glycerophosphocholine (GPC), which can react with a fatty acid or with its vinyl ester to produce Lyso-PL [27]. Immobilized lipases such as *Candida antarctica* lipase B (Lipozyme RM-IM^®^ and Novozym 435^®^) can also be used to convert GPC to LPC, with a conversion yield of 68% and 42%, respectively [38]. However, this method tends to produce mostly LPC with saturated FA such as capric acid (C10:0), lauric acid (C12:0), myristic acid (C14:0), and palmitic acid (C16:0) [39]. Despite its effective reaction, the low reaction yield of this method is a major drawback. To overcome this limitation, GPC esterification was conducted in a solvent-free system since water activity regulation is essential to achieve enzymatic reactions effectively [40]. Another method that was investigated for the synthesis of LPC involves the lipase-catalysed alcoholysis of PC, where alcohol serves as both a substrate and a solvent for the reaction. Lipozyme RM-IM^®^, Lipozyme TL-IM^®^, and Novozym 435^®^ were found to catalyse the conversion of PC in the presence of ethanol, with high LPC yields (>90%), which were obtained using all three lipases [41]. In addition to LPC, fatty acid esters are produced by the reaction as a by-product, which might have some industrial applications and can improve the economics of production. Another research reported the chemical synthesis of 2-LPC from D-mannitol, (*S*)-glycidol, glyceric acid, *p*-nitrophenylglycerate, 3-*p*-toluenesulfonyl-*sn*-glycerol, through several reaction steps, but low yields were also reported [42]. Overall, LPC biosynthesis appears to be the best approach in terms of potential commercial applications.

## 3. Properties of LPC

**Figure 4:**
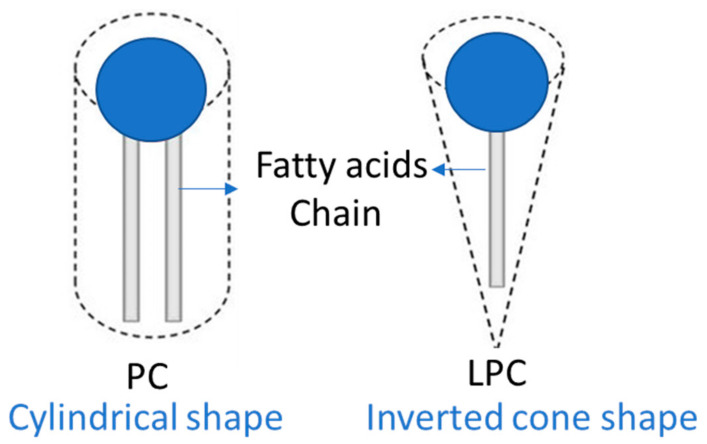
Shape of phosphatidylcholine (PC) and lysophosphatidylcholine (LPC).

The physical and chemical properties of LPC are dependent on its molecular shape, physicochemical properties, and interaction with other compounds in a biological matrix. PCs contain a similar width of hydrophobic tail and polar head group, giving them a cylindrical shape that helps form flat monolayers (Figure 4). In contrast, LPCs have a single fatty acid tail and an inverted cone shape, which helps form micelles with the polar head facing outward (Figure 4). 

Pure LPC is a white solid at an ambient temperature and does not have any characteristic flavour. Biological matrices, including marine organisms, terrestrial animals, and plant tissue, contain LPC as lecithin, which is mainly a mixture of various phospholipids and glycolipids. The colour of LPC containing lecithin is yellow to brown. LPC is an amphiphilic molecule that creates micelles when it disperses in water. The solubility of LPC at 25 °C has been reported in different organic solvents, including ethanol (3.9 mg/mL), methanol (14.6 mg/mL), chloroform (27.8 mg/mL), diethyl ether (0.002 mg/mL), and petroleum ether (0.02 mg/mL) [43,44]. The solubility of LPC was reported to be higher when a mixture of organic solvents, chloroform: methanol (2:1), was used, compared to its solubility in methanol or chloroform alone [45]. LPCs are metabolically stable compared to the parent PC compound [46]. Pure LPC from egg yolk at −0 °C has been reported to be stable for up to 1 year [45].

## 4. Marine Based LPC Compared to Terrestrial Based LPC

Marine-based LPCs are more bioavailable for brain health compared to terrestrial LPCs due to differences in the associated omega-3 content. Marine sources contain long-chain omega-3 PUFAs, including LPC oleate, LPC palmitate, LPC-EPA, LPC-DPA, and LPC-DHA, which can cross the blood–brain barrier (BBB), but LPCs with less than 14 carbons cannot [3,6]. Marine LPC provides dual benefits as a source of both omega-3 fatty acids and phospholipids. For example, after crossing the BBB, LPC-DHA not only increased the DHA level in the brain but also the total phospholipid content. Experimental studies have shown that feeding normal rats with 10 mg of LPC-DHA per day increased both DHA and phospholipid levels in the brain [14]. By contrast, terrestrial-based phospholipids generally contain lower levels of long-chain omega-3 PUFAs and could be less effective in providing the same health benefits. Therefore, marine phospholipids are considered to be a preferred source of omega-3 and phospholipids for supporting brain health. However, the risk of oxidation is higher in marine LPC compared to terrestrial LPC due to higher levels of MUFA and long-chain omega-3 fatty acids in marine sources. To mitigate this risk, marine LPCs should be stored under liquid nitrogen or argon or at least below −20 °C [47]. Due to the presence of the PC head group, both marine and terrestrial LPCs are highly hygroscopic, causing them to alter in structure and have a visible appearance as a gummy or waxy material [45]. Marine LPC oxidation results in the formation of undesirable changes in colour, unpleasant sensory attributes, and secondary oxidation products such as hydroperoxides, ketones, and aldehydes [8,48]. Therefore, protecting marine LPCs from moisture, air, temperature, and the conditions that cause oxidation is critical to improving the storage stability and shelf life of LPCs.

## 5. Factors Affecting Phospholipid Composition

In nature, LPs are present within the lipid matrix and are mostly available as lecithin (a complex mixture of phospholipids and glycolipids) in fish, eggs, soyabean, and other organisms [49,50,51,52]. Various factors, including environmental conditions, sex, age, season, water temperature, pH, and processing conditions, can substantially impact the PL content and its extraction in animals, fish, and other organisms, and, subsequently, the levels of LPC (lysophosphatidylcholine) obtained [49,53]. Given the above comments that marine-sourced PLCs are the most bioavailable, the focus of the present article is on PL from marine sources, and therefore, the information presented will be limited to these resources. Many authors have reported a variation in the phospholipid levels present in fish species. Moreover, the phospholipid composition is also substantially different among different marine species. According to Nanda and Sahu [54], the total phospholipid content of different fish samples collected from freshwater sources ranged from 28.07% to 44.53% of the total lipid in carnivorous and plankton feeder species, respectively. The authors also reported that PC was the predominant phospholipid fraction, followed by phosphatidylethanolamine (PE) in all fish samples. A wider range (6.97–94.6% of total lipid) of phospholipid content in 18 fish species was reported by Drazen [55]. However, Moradi, Bakar, Motalebi, Syed Muhamad, and Che Man [49] mentioned a higher ratio of phospholipids to total lipid content in lean fish, which could reach 90%, compared to that of fatty fish. Regarding the phospholipid classes, Lu, Nielsen, Timm-Heinrich, and Jacobsen [5] revealed the predominance of PC in several marine species, such as salmon, tuna, and rainbow trout, with a range of 37.9% to 54.7% in total phospholipids. PE was the second most predominant fraction of phospholipids in marine sources, which is in agreement with freshwater fishes [55]. Tuna species were reported to have high amounts of sphingomyelin. On the other hand, Gowda, Minami, Gowda, Chiba, and Hui [50] investigated the lipidome of 11 common fish and reported high levels of glycerolipids in shishamo smelt, Japanese sardine, and red seabream, whereas higher glycerophospholipid contents were found in mottled skate, skipjack tuna, and Okhotsk atka mackerel. High levels of sphingolipid fraction were observed in blackfin flounder and flatfish. Phosphatidylcholine was reported to be the major lipid fraction in muscle tissues of four freshwater fish species (Common carp, Mirror carp, Largemouth bass, and Tilapia) collected from Lake Naivasha, Kenya [56]. The contents of phosphatidylcholine and phosphatidylethanolamine in the muscle tissues of the four species were reported to be in the range of 17.78–25.69% and 14.18–21.26%, respectively. The phospholipid content and classes were determined in many species of bony fish (marine and freshwater) and shellfish commonly found in the Italian market by Boselli et al. [57] noted that differences in the phospholipid classes, including phosphatidylcholine, phosphatidylinositol, and phosphatidylethanolamine, could be used to distinguish between shellfish and bony fishes. Phospholipids play vital regulatory and structural roles in fish, and changes in their composition (both qualitative and quantitative) can occur in both the major and minor phospholipid fractions, as well as in their fatty acid composition, in order to maintain the functionality of the cell membrane under varying temperature and hydrostatic pressure conditions experienced by fish at different sea depths. Therefore, such changes may serve as a potential indicator of the environment and habitat of different fish species. Voronin et al. [58] reported phospholipid contents in the range of 0.23% to 2.14% DW in beaked redfish (*Sebastes mentella*) obtained from different depth levels in the Irminger sea. Furthermore, they documented a negative correlation between phospholipid fractions (phosphatidylcholine, phosphatidylethanolamine, phosphatidylserine, and phosphatidylinositol) and the depth at which the fish were located. The fish maturity, size, gender, and origin of the fish can affect the composition and the total phospholipid content of the lipid. For example, farmed small and large-size Bouri Fish (*Mugil cephalus*) collected from a fish farm at Kafr El-Sheikh in Egypt were reported to have a different phospholipid content, ranging from 1.04 to 3.56% of the lipid content, respectively [59]. Additionally, Voronin, Nemova, Ruokolainen, Artemenkov, Rolskii, Orlov, and Murzina [58] reported variations in the number of total phospholipids (0.44–2.14 and 0.23–1.39% DW), major (phosphatidylcholine (0.76–1.6% and 0.16–0.98% DW) and phosphatidylethanolamine (0.12–0.53% and 0.06–0.28% DW)), minor (lysophosphatidylcholine (0.04–0.09 and 0.01–0.07% DW), phosphatidylinositol (0.01–0.06 and 0.003–0.05% DW), and phosphatidylserine (0.01–0.02 and 0.002–0.02% DW) phospholipid fractions between male and female beaked redfish (*Sebastes mentella*), respectively. Moreover, Drazen [55] reported significant (*p* > 0.05) differences in the contents of lysophosphatidylcholine, phosphatidylethanolamine, and phosphatidylserine in the muscles of wild and farmed beaked redfish. Keriko, Chege, Magu, and Githua [56] reported higher amounts of phosphatidylcholine and phosphatidylethanolamine in the muscle tissues of common carp, mirror carp, and largemouth bass compared to their levels in the liver and pyloric caecum tissues. Similarly, muscle tissues of 18 demersal fish species were reported to have higher levels of phospholipids compared to those determined in the liver [55]. Furthermore, samples taken from different areas of farmed beaked redfish varied significantly (*p* > 0.05) in terms of phosphatidylcholine, which was in the range of 0.5–1.7% DW. Fish are mostly consumed after cooking, and thus, it is worth examining the effects of various thermal treatments on the phospholipid content and composition. Given the diversity in cooking styles, it is expected that changes caused by cooking will vary depending on the cooking methods, the associated conditions used (i.e., temperature, dry or wet, the presence of other additives/ ingredients), fish type, and composition [49,60]. When investigating the influence of deep-frying cooking on PE and PC phospholipid classes of *Sardina pilchardus* fillets, researchers found that frying caused significant (*p* > 0.05) changes in the PE and PC molecular species composition of fish fillet [60]. However, frying affected the PC composition more than that of PE. Furthermore, the type of frying oil (extra virgin olive oil, sunflower oil, or high-oleic acid sunflower oil) also impacted the phospholipid composition, whereas it was not affected by the frying temperature (160 and 180 °C) [60].

The deep frying of various fish species (rainbow trout, golden trout, sea bass, herring, coley, and plaice) caused a reduction in the polar lipid content of these fish compared to the fresh ones. This reduction varied from 17 to 50.2% for golden trout and Coley, respectively. Polar lipids were reported to hydrolyse under frying conditions or partition into the frying oil [61], which explains the reduction in their content remaining in fish after frying. The frozen storage of pre-spawned hake fish (*Merluccius hubbsi* Marini) caused a reduction in the phospholipids during the storage process due to enzymatic hydrolysis by phospholipase [62]. Many authors have reported phospholipase activity during the frozen storage of several kinds of fish (rainbow trout, bigeye tuna, chum salmon, mackerel, carp, and Pacific cod) at various storage temperatures [63,64,65]. According to Roldan [62] and Hanaoka and Toyomizu [65], freezing carp fish accelerated the degradation of phosphatidylcholine. Similarly, freezing has been reported to increase the degradation of phospholipids in cod fillets Fauske et al. [66]. However, Roldan [62] reported no significant (*p* > 0.05) change in the total phospholipid content of pre-spawned hake muscles during frozen storage at −20 °C for 250 d. Egyptian traditional salted-fermented Bouri fish (*Mugil cephalus*) has been reported to have a lower phospholipid content compared to fresh fish due to the enzymatic hydrolysis of phospholipids during the fermentation process [60]. Regarding the phospholipid classes, a reduction in the levels of PC, PE, PI, and SM was observed in fermented fish, with an increase in the amount of phosphatidic acid and LPC after fermentation [59]. Similarly, a reduction in the phospholipid molecular species present in Chinese traditional low-salted-fermented carp fish (*Cyprinus carpio* L.) was also reported after the natural fermentation process due to the degradation of phospholipids by both enzymatic and non-enzymatic hydrolysis processes [67].

## 6. Extraction and Quantification of LPC

The extraction of LPC from biological samples involves several steps, including the extraction of the total lipid, the fractionation of the lipid class, the separation of phospholipid as lecithin, and the generation of LPC, using lipase or another suitable hydrolysis method. Phospholipid could be extracted using various extraction methods such as an organic solvent (single solvent or multi-solvent extraction), solid-phase extraction, super/subcritical extraction, pulsed electric field extraction (PEF), enzyme-assisted extraction (EAE), ultrasound-assisted extraction and microwave-assisted extraction [21]. The use of PEF and EAE as aid methods have been reported to reduce the amounts of solvents used for the extraction and to reduce cost as well; they generate bioactive low molecular weight peptides in the lipid fractions, resulting in a higher yield of the total lipid and phospholipid, compared to solvent-only extraction systems [21,68,69,70]. Various fish co-products, including the head, skin, roe, and gonad of fish species commercially fished in New Zealand, such as blue mackerel, hoki, snapper, gurnard, and salmon, have been reported to contain a considerable amount of lysophospholipids [71,72,73], which could be extracted using a one-pot extraction system, ETHEX, which avoids the differential partitioning of lysophospholipids into the aqueous/solvent phase, as occurs in the Folch method [72]. With the ETHEX method, the lipid is extracted from the sample using ethanol/hexane (2:1 *v*/*v*) and high-speed homogenization, followed by filtration and partitioning between the hexane and ethanol phases, which are fractionated using a separation funnel [72]. The phospholipid-containing ethanol phase is evaporated using a rotary evaporator, and the solid lipid fraction is washed with hexane (~50 mL) and acetone (~120 mL). The solvent mixture containing the lipid fraction is centrifuged (15,000× *g*, 15 min) at 4 °C with the resultant pellet containing mostly phospholipids and lysophospholipids. This method is more effective for lysophospholipid extraction compared to the Folch method [74].

Tsushima et al. [47] extracted the total lipid from Japanese common squid skin and North Pacific starfish internal organs using the Bligh and Dyer lipid extraction method [75], and the obtained lipid was passed through a PSQ 60B silica gel column (3 × 30 cm) equilibrated with chloroform/methanol (2:3, *v*/*v*) at 10 mL/min flow rate. The solvent was collected and analysed by thin-layer chromatography to separate and identify the phospholipid classes. Chloroform/methanol/water (65:25:4, *v*/*v*) was used as a mobile phase to separate the PC. A PC band rich in DHA-PC and EPA-PC was collected and mixed with 100 mL hexane and Lipozyme for partial hydrolysis to produce LPC. The headspace of the reaction vessel was filled with argon gas to prevent the oxidation of the generated LPC, while the reactor was shaken for 8 h at 75 strokes/min at 40 °C. With this method, DHA-PC and EPA-PC were converted into DHA-LPC and EPA-LPC, respectively. The produced compounds were reported to suppress angiogenesis [47].

Various quantification methods are used for the determination of phospholipids and lysophospholipid from various biological samples, including thin layer chromatography (TLC), high-performance thin layer chromatography, high-performance liquid chromatography (HPLC), nuclear magnetic resonance spectroscopy (NMR), supercritical fluid chromatography (SFC) and vibrational spectroscopy (e.g., FT-IR). The TLC method is the earliest chromatographic method that has been developed for the separation and analysis of various lipid classes, in which an organic solvent mixture is used as a mobile phase, and cellulose or silica gel is used as the stationary phase as a thin layer applied to a backing plate of glass or aluminium. The TLC method is relatively easy to conduct, reliable, inexpensive, and requires minimal sample preparation. There are two different formats of TLC, 1-dimensional TLC (1D-TLC) and 2-dimensional TLC (2D-TLC). 1D-TLC is less sensitive for the detection of lysophospholipids compared to 2-D TLC [76]. For instance, 1D-TLC (mobile phase: methanol, glacial acetic acid, and water: 50:8:8:2, *v*/*v*; stationary phase: silica gel plate) has been reported to detect only major phospholipids (PE, PC, and PI) in salmon co-products [76]. Another study detected most of the phospholipids and lysophospholipids in dairy lipid samples using 2D-TLC [76].

TLC can be a useful technique when used as a preliminary qualitative method, but it has its limitations in terms of accuracy, resolution, and reproducibility. However, these limitations can be overcome by utilizing advanced techniques such as HPLC, LCMS, or NMR, which offer higher levels of precision and reproducibility. The HPLC and LCMS techniques are the most robust and accurate and are used for the quantitative determination of phospholipids and lysophospholipids that are present in a complex lipid mixture. However, sample preparation involves a series of steps that require technical skills and toxic organic solvents. This process is also not suitable for the simultaneous quantification of phospholipids and lysophospholipids under industrial conditions, for which NMR and vibrational spectroscopic methods (FT-IR and FT-Raman) offer advantages compared to chromatographic techniques. Both NMR and vibrational spectroscopic methods are eco-friendly, require minimal or no sample preparation, are rapid, non-destructive, and can be used online/inline/offline in an industrial process for the simultaneous detection and quantification of phospholipid and LPC. ^31^P (phosphorus) NMR is a well-known technique for the detection of phospholipids in various biological samples, including fish co-products [71,72,73], algae [77], and milk phospholipids [76,77,78]. FT-IR was suggested as an alternative to ^31^P NMR for the quantification of phospholipids in krill oil [79]. Various methods of phospholipid extraction and quantification have been comprehensively reviewed elsewhere [21], and an interested reader is directed to consult these resources.

## 7. Neuroprotection and Brain Health Application of LPC

As previously mentioned, LPCs are the most abundant LPC in the blood, and they are associated with albumin and lipoproteins. Blood LPC levels decline with age, which was found to be a predictor of memory impairment in the elderly [80]. Additionally, LPC-DHA is the lyso-PL form commonly found in the brain compared to other organs, such as the liver, retina, heart, and kidneys [24]. Previous research has emphasized the fundamental role of LPC containing omega-3 PUFA, mainly DHA (C22:6 omega-3) and eicosapentaenoic acid EPA (C20:5 omega-3), in brain development and neuronal cell growth [16,81,82]. Research has shown that LPC-DHA/EPA-enriched krill oil intake exhibited neuroprotection activity and slowed down dementia-related behaviour in mice, which is associated with cognitive degeneration and the hazards of age-correlated neurological diseases [16]. A decline in APOE4 could be related to low levels of DHA; hence, several studies have proposed that long-term treatment with LPC-DHA/EPA can reduce the severity of neurodegenerative disorders [16,81,82,83,84].

More recently, the neuroprotective role of LPC-DHA supplementation on the brain post-cardiac arrest (CA) has been suggested [85]. A decreased level of LPC-DHA in the plasma of CA human patients was strongly linked to neurological deficiencies and the loss of difference between grey and white matter in the brain. LPC-DHA supplementation improved brain levels of LPC-DHA and protected neurons and astrocytes in the brain [85].

Furthermore, a therapeutic prospective of engineered brain-targeting forms of omega-3 PUFA for acute stroke treatment, called AceDoPC^®^, was evaluated in vivo [86]. The neuroprotection effect of AceDoPC^®^ was demonstrated in an experimental rat stroke model that had undergone intraluminal middle cerebral artery occlusion (MCAO) treatment, which was investigated using multimodal MRI [86]. The results obtained indicated that the neuroscores of AceDoPC^®^-treated rats were inferior compared to that of DHA-treated rats and that both DHA and AceDoPC groups had considerably reduced lipid peroxidation in comparison to pooled saline and vehicle control groups. Moreover, an MRI-centred estimation established the neuroprotective effect of DHA and AceDoPC^®^ in the MCAO model [86]. The same experiments could be performed with LPC-DHA since AceDoPC^®^ is considered to be a stabilised form of LPC-DHA.

LPC-EPA/DHA could be used to support treatment programs for depression, since improvements in cerebral EPA and DHA contents were observed in subjects fed a diet high in LPC-EPA [15]. Additionally, in the brain, a high expression of the brain-derived neurotrophic factor (cyclic AMP response element binding protein) and serotonin receptor (5-hydroxy tryptamine1A) were detected. Since LPC-EPA enhanced cerebral EPA and DHA levels, it may support the treatment of depression [15].

However, there are reports on the controversial effects of LPC, where both pro-inflammatory and anti-inflammatory activities have been reported [87]. Under certain physiological conditions, it has been found that the LPC concentration in plasma can increase substantially under some inflammatory conditions [88]. However, this is not always the case since the LPC/PC ratio in plasma, as well as in cerebrospinal fluid from patients with Alzheimer’s disease (AD), was found to decline [89,90]. In comparison to healthy subjects, the brains of AD patients have lower levels of LPC, which is associated with a greater risk of mortality [91]. LPC supplementation is reported to improve brain function in various model systems (Table 1).

Less information is available in the literature about the neuroprotective potential of LPC and its ability to reduce oxidative stress in the brain. Since LPC is the preferred transporter of DHA into the brain, as previously discussed, it would be beneficial to elaborate more regarding DHA metabolism and the production of metabolites that might decrease the release of reactive oxygen species (ROS). Indeed, DHA can be oxidised by numerous lipoxygenases (LOX) [92]. Mono-, di- and tri-hydroxy-metabolites of DHA have been identified. The lipoxygenation of DHA via 15-LOX results in the production of protectin D1 (PD1): a member of the class of specialized pro-resolving mediators. PD1 was found originally in activated mononuclear cells and in the brain, where it was called neuroprotection D1 (NPD1) and was suggested to provide neuroprotective and anti-inflammatory effects in tissues [93,94]. The PD1/NPD1 mainly inhibited neuronal apoptosis associated with neurodegeneration in AD [93,94]. Researchers discovered an isomer of PD1, called PDX, with conjugated triene geometry E/Z/E for PDX and E/E/Z for PD1. PDX was reported to act as an inhibitor of cyclooxygenase 1 (COX-1) and cyclooxygenase 2 (COX-2) [95]. In vitro, PDX reduced the production of ROS by human neutrophils [95]. PDX is considered an anti-inflammatory and anti-aggregation agent that inhibits ROS production in the brain. Therefore, it is possible that targeting the brain with LPC-PDX, where PDX is esterified at the *sn*-2 position, could enhance these activities. However, further research is needed to confirm this hypothesis.

**Table 1:** Effect of LPC supplement on brain health.

Types of PL Form	Model System	Treatment and Duration	Findings	References
1-DHA-LPC 2-DHA-LPC	adult mice	1 mg/d (oral administration); 1 month	-Increased the percentage of DHA and C20:4 in the plasma -Improved DHA level and C22:6/C20:4 ratio in all regions of the brain -LPC-DHA treatment remarkably improved memory and spatial learning -Increased molecular species of DHA-PC, DHA-PE in the plasma and BNDF level (brain derived neurotrophic factor) in all brain regions	[14]
*sn-1* LPC-EPA	adult mice	1 mg DHA per d or ∼40 mg/kg body weight	-Improved memory and cognition. -LPC-EPA in the brain increased from 0.03 to 4 μmol/g (>100-fold) -Little effect on free EPA -DHA was increased 2-fold by LPC-EPA but not by free EPA. -LPC-EPA also increased DHA concentration in the retina and improved brain-derived neurotrophic factors in the brain	[15]
Labelled LPC	adult squirrel monkeys	1.36 μmol	-LPC in the plasma is taken up by the brain, metabolized in brain tissue and acts as a precursor of PC and choline	[96]
LPC-DHA	APOE3- and APOE4-TR mice	4 to 12 months	-LPC-DHA-enriched krill oil could increase DHA level in the brain -Improved memory relevant behaviour -LPC-DHA supplements could be preventive for some level of age-related neurodegeneration	[16]
LPC	adult mice	-	-Revealed the protection against LPC-induced demyelinating lesion and cognitive deficits -Could be a feasible and promising therapeutic treatment against demyelinating diseases	[97]
LPC-DHA	expectant mice	DHA-LPC (68.8 mg/mL, oral administration); 4 d	-Improved the DHA level in the brain	[98]

## 8. Role of LPC in Drug Delivery

Due to its high levels in the blood, LPC was recommended as a carrier of FA (mainly omega-3 PUFA) and choline to the brain and other tissues. The role of LPC in drug delivery is summarized in Table 2. Thiés et al. [27] investigated whether unsaturated 2-acyl-LPC linked to plasma albumin was an effective carrier of unsaturated FAs in the brain using 20-day-old rats, with comparison to non-esterified forms of these fatty acids [28]. Labelled ^14^C palmitic (C16:0), ^14^C oleic (C18:1n-9), ^14^C linoleic (C18:2n-6), and ^14^C arachidonic acids (C20:4n-6), in either their non-esterified form or esterified in LPC labelled with ^3^H on the choline and ^14^C fatty acid groups, were perfused for 30 s. The authors suggested that 2-acyl-LPC was an efficient delivery form of unsaturated FAs to the rat brain and that the FA delivery form could control their fate in tissues. The same research team studied the uptake and metabolism of ^3^H-DHA when esterified at the *sn-2* position of lysophospatidylcholine-DHA (^3^H-LPC-DHA), and in a non-esterified form, in 20-day-old rats, through the perfusion of 100 µL of lipid solution with 1 µCi ^3^H-DHA-LPC (12 nmol) into the tail vein for 30 s [13]. They showed that LPC-^3^H-DHA was favourably deposited in the brain (4–5% of injected radioactivity) compared to the non-esterified form of DHA (0.3–0.4%), thus suggesting that the developing brain took up *sn-2*-LPC-DHA more than unesterified DHA [13].

The accretion of DHA esterified at the *sn-2* position of LPC-^3^H-DHA in comparison to the non-esterified form of ^3^H-DHA was investigated using an in vitro model of BBB [29]. LPC-^3^H-DHA was found to be a favoured functional carrier of DHA to the brain when compared to non-esterified ^3^H-DHA. Sugasini et al. [14] showed that supplementation of DHA from triacylglyecrol (TAG) or from natural PC (where DHA is esterified at the *sn-2* position) was not suitable to enhance DHA content in the brain, whereas DHA from LPC (at the *sn-1* or the *sn-2* position) efficiently enhanced the DHA level in brain [14].

Furthermore, Hachem et al. [33,99] conducted in vitro, in vivo, and ex vivo studies to examine the cerebral accretion of a stabilized form of LPC-DHA named AceDoPC^®^ (1-acetyl, 2-docosahexaenoyl-glycerophosphocholine) in comparison to other forms of DHA. The in vitro study investigated the passage of non-esterified ^14^C-DHA, ^14^C-PC-DHA, and ^14^C-AceDoPC^®^ across a model of the blood–brain barrier (BBB) in triplicates (0.1 μCi, 5 μM per molecule), whereas the in vivo study examined the effects of ^14^C-DHA, ^14^C-PC-DHA or ^14^C-AceDoPC^®^ injected into 20-day-old rat tail veins (0.5 μCi, 1 mM injected). The rats were sacrificed at 15 min, and 1, 6, 24, and 48 h after injection. The brain, heart, liver, eyes, and blood were collected, and lipids were extracted. The results from the in vitro and in vivo studies reported for the first time that AceDoPC^®^ was a privileged and precise carrier of DHA to the brain, not in the other studied organs when compared with DHA containing PC and non-esterified DHA. The research reported that AceDoPC^®^ was hydrolysed, in part, into LPC-DHA [33]. The formation of LPC-DHA may be due to the hydrolysis of the acetyl moiety by the action of the endothelial lipase, a phospholipase A_1_ (PLA_1_)-like enzyme that has been shown to be expressed and secreted in brain capillary endothelial cells (BCECs) and to have a preference for DHA-containing phospholipids at the *sn-2* position [100].

Ex vivo, the bioavailability of ^13^C-DHA in the elderly (60–70 years old subjects), after oral ingestion of a single dose (50 mg) of ^13^C-DHA, whether non-esterified or esterified in ^13^C-AceDoPC^®^, or esterified in triacylglycerol (^13^C-TAG-DHA), was studied [33,99]. Gas chromatography/combustion/isotope ratio mass spectrometry (GC/C/IRMS) was used to evaluate the ^13^C enrichment of DHA-containing lipids. This study suggests that DHA from AceDoPC^®^ was more effective than TAG-DHA for a continuous increase in red cell ethanolamine phospholipids connected to improved brain accretion [33,99]. The same clinical study could be applied with LPC-DHA instead of AceDoPC^®^ to transport DHA to the brain.

Concerning the mechanisms involved in LPC-DHA transport to the brain through the BBB, the study by Nguyen et al. [11] highlighted the acute function of a major facilitator superfamily domain-containing protein 2 (Mfsd2a), expressed in BBB endothelium, as a chief transporter for LPC-DHA uptake to the brain. Mfsd2a is a membrane transport protein expressed in the endothelium of the BBB and plays a vital role in BBB development and functions [11]. For example, *Mfsd2a* knockout mice indicated a 60% decline in cerebral DHA, cognitive impairment, and neuronal damage in the hippocampus [11]. Additionally, LPC-oleate and LPC-DHA levels in the brain decreased by 80% and 90%, respectively, in *Mfsd2a* knockout mice compared to wild-type mice. *Mfsd2a* knockout mice had a 30% lower brain weight than wild-type mice. A free DHA diet did not increase brain weight or brain DHA in the *Mfsd2a* knockout mice. Contrarily, DHA levels in the heart and liver were not considerably different between *Mfds2a* knockout and wild-type mice groups, suggesting that *Mfsd2a* is a key component in PUFA transport to the brain but not to other organs [11]. Other researchers investigated the lack of a *Mfsd2a* receptor and suggested the dynamic role of LPC-DHA in developing the brain [101,102].

## 9. LPC for the Delivery of Other Bioactive Ingredients

Lysophosphatidylcholine (LPC) has been recently explored as a potential nano-carrier for the delivery of neuroprotective drugs. LPC is naturally found in the body, and its physicochemical properties make it a promising candidate for drug delivery systems [103]. Recent studies have shown that LPC-based delivery systems can improve the properties and efficacy of neuroprotective drugs [104]. For example, LPC was used as a carrier for the delivery of honokiol: a natural compound with neuroprotective effects [105]. The researchers found that honokiol-loaded LPC nanoparticles exhibited better solubility, stability, and sustained release compared to free honokiol. Moreover, the nanoparticles were able to cross the blood–brain barrier and accumulate in the brain, resulting in significant neuroprotection against oxidative stress-induced injury in rats. Another study investigated the use of LPC-based nanocarriers for the delivery of edaravone: a drug used for the treatment of acute ischemic strokes [106]. The researchers found that the nanocarriers improved the solubility and stability of edaravone and enhanced its neuroprotective effects in a rat model of ischemic stroke. Moreover, the nanocarriers were able to target damaged brain tissues and release the drug in a sustained manner, resulting in prolonged therapeutic effects. LPC-based delivery systems have also been shown to improve the bioavailability and efficacy of other neuroprotective drugs, such as curcumin, resveratrol, and quercetin [107]. These compounds are known for their antioxidant, anti-inflammatory, and anti-apoptotic effects, which make them promising candidates for the treatment of neurodegenerative diseases. However, their poor solubility and bioavailability limit their clinical applications. LPC-based nanocarriers have been shown to overcome these limitations by improving the solubility, stability, and targeted delivery of these compounds to the brain.

LPC-based delivery systems also provide the opportunity to improve the properties of some bioactive compounds during storage and absorption. One delivery system that may benefit from the presence of LPC is liposomal encapsulation. The use of LPC for the manufacture of liposomes has been known for more than four decades. In 1980, in an attempt to enhance the delivery of liposomal contents into cells, Ralston et al. [108] investigated the effect of LPC on the transfer of carboxyfluorescein from both small unilamellar vesicles (SUVs) and large multilamellar vesicles (LMVs) to human lymphocytes. It was found that in the presence of LPC, the SUVs were smaller, and the LMVs had less leakage, but LPC did not exert any significant effect (*p* > 0.05) on the transfer of carboxyfluorescein to the lymphocytes. A recent investigation [109] used LPC and phosphatidylcholine (PC) for the manufacture of liposomes in order to improve nephroprotective effects and the oral absorption of astaxanthin. The authors reported that at a pH close to neutral (pH 6.8), such liposomes exhibited an improved dissolution behaviour of astaxanthin when compared to the behaviour of untreated/crystalline astaxanthin. Particularly, the release of astaxanthin from the liposome-containing LPC was seven-fold higher than that of those manufactured using PC, besides a significant increase (*p* < 0.05) in systemic exposure to astaxanthin (a 15-fold higher after 24 h after oral administration), when compared to liposomes made of PC. Based on the results obtained from histological analysis and plasma biomarkers in the rat model of kidney injury, the researchers concluded that the LPC-based liposomes exhibited the improved nephroprotective effects of astaxanthin [109]. Therefore, these findings suggest that an LPC-based liposomal approach might provide a sustainable option for improving the bio-efficacy of astaxanthin, which may be applicable to other lipophilic bioactive ingredients as well. In another recent study, Li et al. [110] used LPC to engineer liposomes and improve the uptake efficiency of LPC in tumour cells (4T1 cells).

Another bioactive delivery system that may benefit from the presence of LPC is an emulsion system. For example, the preparation and characterization of oil-in-water nano-emulsions containing curcumin and using LPC as an emulsifier have been studied [111]. The findings from that research showed that the addition of LPC resulted in nano-emulsions with particle sizes of less than 500 nm, while there was no significant (*p* > 0.05) change in this parameter (i.e., original droplet size) in the polydispersity index, or in zeta potential values over the storage of 12 weeks.

## 10. Safety of LPC

Although many studies have reported the importance of LPC in brain health as a carrier of DHA [13,16,29] exerting positive effects with several other pathologies [23], many studies have highlighted negative associations between LPC and diseases. For example, LPC has been reported to exhibit pro-inflammatory, cytotoxic, and anti-haemostatic activities in various model systems [23,112]. The pro-inflammatory activity of LPC was demonstrated by stimulating the production of reactive oxygen species and the release of chemotactic factors (compounds that stimulate the movement of cells in a gradient) that play a major role in inflammation [113,114]. The pro-inflammation effects of LPC appear to be dependent on its concentration [114] and the fatty acid esterified to the LPC [23]. For example, saturated fatty acids (C16:0 and C18:0) and the monounsaturated fatty acid C18:1 esterified in LPC, exert a pro-inflammatory response due to their production of radical species as a result of oxidation, whereas long-chain fatty acids such as ardenic acid (C22:4) and cervonic acid (C22:6) exhibit anti-inflammatory activity. Acute inflammation was found to be caused by LPC injection in healthy subjects [115] and was accompanied by the substantial build-up of monocytes, T lymphocytes, and neutrophils resulting from the treatment. Excess LPC was found to induce the production of cytokines in the smooth muscle, which caused atherogenesis and impaired the vasodilation of the aorta [116]. These two events are triggered by the involvement of LPC in the oxidation of low-density lipoprotein and the inhibition of acetylcholine, respectively. Other studies have reported increased oxidative stress as a contributing factor due to the high production of nitric oxide in the presence of high LPC concentrations [117,118]. Using various cell models, significant biochemical events were observed due to elevated LPC content. For example, using endothelial cells, many studies demonstrated the overexpression of the neurogenic locus notch homolog protein 1 (Notch 1), the Hairy and Enhancer of split proteins (HES1), monocyte chemoattractant protein-1 (MCP-1), induced cytotoxicity and apoptosis, the production of interleukin 8 (IL-8), and inhibition of endothelial cell migration and proliferation [115,117,119,120,121]. Using adipocytes and immune cells, several studies have demonstrated the overproduction of IL-1, IL-6, and tumour necrosis factor (TNF) by adipocytes [122,123] and induced the production of TNF and TNF while activating macrophages, B cells, and transforming growth factor 1 (TGF-1) production in immune cells [124,125,126,127]. Furthermore, LPC has been reported to cause substantial damage to the gastric mucosa and leads to extensive injury [128]. An elevated LPC concentration in the plasma has been linked to several diseases (cardiovascular, diabetes, cancer, and renal failure [26,112], and LPC can be used as a biomarker for a wide range of diseases, especially those that are age-related [112]. A possible strategy is to reduce LPC levels and eliminate its production via the PLA_2_ pathway [88], which could reduce LPC production and reduce the oxidation of long-chain fatty acids that generate free radicals.

## 11. Conclusions

In summary, LPCs are important for brain development and protection. Supplementing with LPC-DHA/EPA has been associated with improved brain function, reduced neurological deficiencies, and potential treatments for depression and post-cardiac arrest. Although research is ongoing to fully understand their neuroprotective potential, the metabolism of DHA and the production of its metabolites involving PD1 and PDX may provide key insights into the beneficial effects of LPCs. While some negative associations have been found with diseases such as cardiovascular disease, diabetes, cancer, and renal failure, the positive effects of LPC as a carrier of DHA in various studies cannot be overlooked. Additionally, the anti-inflammatory, anti-aggregating, and ROS-inhibiting properties of LPC-PDX make it a promising agent for targeting the brain. Thus, LPCs have great potential for improving brain health, but their safety and potentially negative effects should also be taken into consideration.

## Figures and Tables

**Table 2:** Role of LPC in drug delivery.

Study	Objective	Method	Result
Thiés et al. [13]	To investigate the efficacy of 2-acyl-LPC linked to plasma albumin as a carrier of unsaturated FAs to the brain	Perfusion of 20-day-old rats with labeled ^14^C fatty acids in either non-esterified or esterified form in LPC labeled with ^3^H on the choline and 14C fatty acid groups	2-acyl-LPC was an efficient delivery form of unsaturated FAs to the rat brain, suggesting that the FA delivery form could control their fate in tissues
Thiés et al. [13]	To compare the uptake and metabolism of ^3^H-DHA esterified at the *sn*-2 position of LPC-DHA and in a non-esterified form in twenty-day-old rats	Perfusion of 100 µL of lipid solution with LPC containing 1 µCi ^3^H-DHA (12 nmol) into the tail vein for 30 s	^3^H-LPC-DHA was favourably improved in th e brain (4–5% of injected radioactivity) over the non-esterified form of DHA (0.3–0.4%), suggesting that the developing brain favorably employed *sn*-2-LPC-DHA compared to unesterified DHA
Sugasini et al. [14]	To determine the suitability of different sources of DHA supplementation in enhancing brain DHA levels	Feeding 20-day-old rats with DHA from triacylglycerol (TAG), natural PC, or LPC for four weeks	LPC (at *sn*-1 or *sn*-2 position) efficiently enhanced the DHA level in the brain, while DHA from TAG or natural PC (where DHA is esterified at *sn*-2 position) was not suitable for enhancing brain DHA content
Hachem et al. [33,99]	To examine the cerebral accretion of AceDoPC^®,^ a stabilized form of LPC-DHA, in comparison to other forms of DHA	In vitro and in vivo studies investigating the passage of non-esterified ^14^C-DHA, ^14^C-PC-DHA, and ^14^C-AceDoPC^®^ across a model of a blood–brain barrier (BBB) and the effects of the same forms of DHA injected into a twenty-day-old rat tail vein, respectively	AceDoPC^®^ was a privileged and precise carrier of DHA to the brain, not in the other studied organs, when compared with DHA-containing PC and non-esterified DHA

## Data Availability

Not applicable.
